# Changes in Self-Reported Adult Health and Household Food Security With the 2021 Expanded Child Tax Credit Monthly Payments

**DOI:** 10.1001/jamahealthforum.2023.1672

**Published:** 2023-06-24

**Authors:** Jordan M. Rook, Cecile L. Yama, Adam B. Schickedanz, Alec M. Feuerbach, Steven L. Lee, Lauren E. Wisk

**Affiliations:** 1 Greater Los Angeles Veterans Administration Healthcare System, Los Angeles, California; 2 University of California, Los Angeles National Clinician Scholars Program, Los Angeles, California; 3 Department of Surgery, David Geffen School of Medicine at UCLA, Los Angeles, California; 4 Department of Pediatrics, David Geffen School of Medicine at UCLA, Los Angeles, California; 5 Department of Emergency Medicine, State University of New York Downstate Kings County, New York; 6 Division of General Internal Medicine and Health Services Research, David Geffen School of Medicine at UCLA, Los Angeles, California; 7 Department of Health Policy and Management, Fielding School of Public Health at UCLA, Los Angeles, California

## Abstract

**Importance:**

The 2021 Expanded Child Tax Credit (ECTC) provided families with children monthly payments from July 2021 to December 2021. The association of this policy with adult health is understudied.

**Objective:**

To examine changes in adult self-reported health and household food security before and during ECTC monthly payments.

**Design, Setting, and Participants:**

This repeated cross-sectional study used multivariable regression with a difference-in-differences estimator to assess adult health and food security for 39 479 respondents to the National Health Interview Survey (January 2019 to December 2021) before vs during monthly payments. Analyses were stratified by income to focus on low-income vs middle-income and upper-income households.

**Exposure:**

Eligibility for ECTC monthly payments from July 2021 to December 2021.

**Main Outcomes and Measures:**

Overall self-reported adult health and household food security as binary outcomes (excellent or very good health vs good, fair, or poor health; food secure vs food insecure).

**Results:**

In this nationally representative cross-sectional study of 39 479 US adults (mean [SD] age, 41.0 [13.0] years; 7234 [21.7%] Hispanic, 321 [0.9%] non-Hispanic American Indian/Alaska Native, 2205 [5.7%] non-Hispanic Asian, 5113 [13.7%] non-Hispanic Black, and 23 704 [55.8%] White individuals), respondents were predominantly female (21 511 [52.4%]), employed (33 035 [86.7%]), and married (19 838 [55.7%]). Before disbursement of ECTC monthly payments, 7633 ECTC-eligible adults (60.1%) reported excellent or very good health, and 10 950 (87.8%) reported having food security. Among ECTC-ineligible adults, 10 778 (54.9%) reported excellent or very good health and 17 839 (89.1%) reported food security. Following disbursement of monthly payments, ECTC-eligible adults experienced a 3.0 percentage point (pp) greater adjusted increase (95% CI, 0.2-5.7) in the probability of reporting excellent or very good health compared with ECTC-ineligible adults. Additionally, ECTC-eligible adults experienced a 1.9 pp greater adjusted increase (95% CI, 0.1-3.7) in the probability of food security than ECTC-ineligible adults. In income-stratified analyses, the association between ECTC eligibility and overall health was concentrated among middle-income and upper-income households (3.7-pp increase in excellent or very good health; 95% CI, 0.5-6.9). Conversely, the association between ECTC eligibility and food security was concentrated among low-income adults (3.9-pp increase in food security; 95% CI, 0-7.9).

**Conclusions and Relevance:**

The results of this cross-sectional study suggest that monthly ECTC payments were associated with improved adult overall health and food security. Cash transfer programs may be effective tools in improving adult health and household nutrition.

## Key Points

**Question:**

Were 2021 Expanded Child Tax Credit (ECTC) monthly payments associated with changes in adult overall health or household food security?

**Findings:**

In this repeated cross-sectional study using nationally representative survey data from 39 479 respondents and a difference-in-differences design, eligibility for ECTC payments was associated with improved overall adult health and household food security.

**Meaning:**

The results of this cross-sectional study suggest that the COVID 19–era policy of ECTC monthly payments may have been associated with improved health and nutrition in adults in eligible households.

## Teaser

This cross-sectional study examines changes in adult self-reported health and household food insecurity before and during Expanded Child Tax Credit monthly payments.

## Introduction

Among high-income countries, the US has high relative poverty rates, with 1 in 6 families with children with an income lower than the federal poverty level (FPL).^1^ Abundant research has shown an association between poverty and reduced longevity and poorer health and development.^2,3,4^ To address this, many countries have enacted unconditional monthly cash transfer programs for families with children with associated improvements in health outcomes.^5,6,7,8^ In contrast, the US has historically favored smaller annual tax rebates for working families, such as the Child Tax Credit.^6,9^ It is unclear how more generous cash transfer programs might be associated with adult health outcomes in the US.^10,11^

In response to the COVID-19 pandemic, the American Rescue Plan of 2021 made several changes to the Child Tax Credit, including increasing the annual value of the credit to $3600 for each child 5 years or younger and $3000 for each child aged 6 to 17 years.^12^ Half of the total value was dispensed in monthly payments from July 15 to December 31 of 2021. These monthly payments were associated with a temporary 40–percentage point reduction in the rate of poverty in households with children.^13^ Unlike prior iterations of the Child Tax Credit, these payments were fully refundable, meaning that all low-income households were eligible to receive the full value of the credit as a direct deposit or check.^14^ Since expiring at the end of 2021, the Child Tax Credit reverted to its prior design.^15,16^ As one of the largest economic policy interventions in recent history (the US Congressional Budget Office estimated that the 1-year expansion of the Child Tax Credit cost $185 billion^17^), it is vital to understand how the Expanded Child Tax Credit (ECTC) was associated with changes in health to inform the design of future cash transfer programs.

To date, to our knowledge, only 2 studies have assessed the association between the ECTC and adult well-being, with 1 study finding a reduction in depression and anxiety symptoms among low-income adults and the other finding no association between the ECTC and changes in adult life satisfaction or mental health.^18,19^ To our knowledge, no study has examined the association of the ECTC with adult overall health status.

Four studies have investigated the association between the ECTC and household nutrition. However, 3 of these studies only assessed food insufficiency, which is a narrow, single-question measure of household nutrition.^20,21,22^ The only study to address food security, which is often measured with the US Department of Agriculture’s (USDA) 10-question survey, recruited participants from a single medical center and may have limited generalizability.^23^ To our knowledge, no study has assessed the association between the ECTC and food security using a nationally representative sample. Using nationally representative data from the National Health Interview Survey (NHIS), we investigated the association between ECTC eligibility and self-reported overall adult health and secondarily evaluated food security and its potential as a mediator of this association.

## Methods

### Data Source and Sample

We used NHIS responses from January 1, 2019, to December 31, 2021. The NHIS is an annual nationally representative, cross-sectional household survey of the civilian noninstitutionalized population living in the US. Households are selected using geographically clustered sampling, with annual response rates ranging from 48.9% to 59.1%. One adult is randomly interviewed per household and surveyed on health, health care access, and sociodemographic characteristics.^24^ This study followed Strengthening the Reporting of Observational Studies in Epidemiology (STROBE) reporting guidelines for cross-sectional studies.^25^ This study was deemed exempt by the University of California, Los Angeles institutional review board, which also waived informed consent due to use of deidentified data.

We included all households with a sample adult younger than 65 years and with a household income lower than the threshold for full eligibility for the ECTC. This included single adults with a household income less than $75 000 or married adults with a household income less than $150 000.^9,12^ We derived income using the household income to poverty ratio, household size, and the US Census Bureau’s 2020 poverty thresholds (eTable 1 in Supplement 1). Among the 40 175 households for which we could determine ECTC eligibility, we excluded 696 households in which the adult participants were missing any outcome or covariate used for analysis; this resulted in 39 479 households being included in the analytic sample.

### ECTC Eligibility Groups

We separated income-eligible adults into 2 exposure groups based on the presence of any child 17 years or younger in their household, which was consistent with prior studies.^20,21,22^ Households with children were defined as the ECTC-eligible group. Households without children were defined as the ECTC-ineligible group. We used the month and year of interview to compare those surveyed before disbursement of monthly payments in July 2021 (ie, the pre-ECTC group) vs those surveyed during disbursement of monthly payments from July 2021 to December 2021 (ie, the during-ECTC group).

### Self-Reported Overall Health (Primary Outcome)

We evaluated adult self-reported overall health from the survey question, “Would you say your health in general is excellent, very good, good, fair, or poor?” We collapsed responses into a binary variable of excellent or very good health vs good, fair, or poor health, consistent with prior studies.^26,27^ We elected to use the average health of the sample as the cut point for the dichotomization. In the sample, the “average health” state fell between very good and good health. This was consistent with prior studies comparing self-reported overall health with more expansive health utility scales.^28,29^

### Household Food Security (Secondary Outcome)

Household food security was determined using a series of 10 questions designed by the USDA. *Food security* is defined as having “access to enough food for an active and healthy life,” whereas *food insecurity* is defined as “limited availability of nutritionally adequate and safe foods, or limited ability to acquire acceptable foods in socially acceptable ways.”^30,31^ Per USDA scoring recommendations, households with a raw score of 0 have high food security, a score of 1 to 2 represents marginal food security, 3 to 5 represents low food security, and 6 to 10 represents very low food security. These can be further collapsed into a binary outcome, with the former 2 categories comprising food security and the latter 2 categories comprising food insecurity.^24,30^

### Covariates

We designed a conceptual model before statistical analyses to identify socioeconomic and demographic covariates that may alter overall health or food insecurity. We adjusted for the following characteristics in the regression models: age, sex, race and ethnicity, highest household educational attainment, household employment status (any household member employed), marital status, burden of chronic disease, anxiety or depression diagnosis, health insurance, urbanicity, number of household adults, and receipt of Supplemental Nutrition Assistance Program (SNAP) benefits. Race and ethnicity categories included Hispanic, non-Hispanic American Indian or Alaska Native, non-Hispanic Asian, non-Hispanic Black, non-Hispanic White, and non-Hispanic multiracial or Other. Race and ethnicity were self-reported by participants. The NHIS did not describe the racial and ethnic groups included in the category of “Other.” We included receipt of SNAP as a covariate given changes in the benefit associated with the COVID-19 pandemic and to remain consistent with similar prior studies.^20,21,22,32^

### Statistical Analyses

Analyses were performed using Stata, version 17.0 (StataCorp), accounting for the complex sampling design and weighting scheme of the NHIS. All tests were 2-sided, with an α level of .05. We described sociodemographic characteristics by ECTC eligibility and by pre-ECTC vs during-ECTC status. We conducted bivariate analyses between groups using 2-sample *t* tests and χ^2^ tests. We did not adjust the significance level for multiple comparisons for the 2 prespecified outcomes given that the secondary outcome of food security was a confirmatory analysis included to evaluate its potential as a mediator of changes in adult health. The results should be interpreted accordingly.^33,34^

We used linear probability models with difference-in-differences estimators to evaluate changes in overall health and food security for ECTC-eligible households compared with ECTC-ineligible households before and after the initiation of monthly payments in July 2021. We graphed trends for both outcome variables to ensure that the common trends assumption for difference-in-differences analysis was not violated. We found no difference in preperiod trends between ECTC eligibility groups overall or by income level (eFigure in Supplement 1). We derived unadjusted frequencies and adjusted marginal probabilities to describe changes in health status and food security by ECTC eligibility before and after initiation of monthly payments. We compared these values to produce difference-in-difference estimates for each health and food security state. We further stratified the analyses by household income as a percentage of the FPL (≤200% FPL as low-income vs >200% FPL as middle-income and upper-income) to identify if there were differential ECTC effects based on income. We did not adjust for multiple comparisons in these stratified analyses, so the results should be interpreted as exploratory.^33,34^ As we hypothesized that food security may be a potential mechanism by which the ECTC was associated with overall health, we evaluated food security as a mediator via the Sobel method (eTable 2 in Supplement 1).^35^

### Sensitivity Analyses

We conducted several additional analyses to assess the sensitivity of the findings. First, we conducted separate analyses using an income threshold of $112 500 for nonmarried adults instead of $75 000, reflecting the income level at which individuals who file taxes as a head of household vs single are no longer eligible to receive the full ECTC (eTable 3 in Supplement 1).^9^ The NHIS does not provide respondent tax filing status, so we were unable to determine the filing status of nonmarried adults. Second, we evaluated alternative specifications of the modeling approach including logistic regression (eTable 4 in Supplement 1) and ordinal logistic regression (eTable 5 in Supplement 1). Third, as prepandemic responses may have affected the outcomes, we conducted analyses that excluded 2019 data (eTable 6 in Supplement 1). Lastly, we conducted analyses that excluded receipt of SNAP as a covariate given similarities in eligibility between this program and the ECTC (eTable 7 in Supplement 1).

## Results

### ECTC Eligibility Group Characteristics

The study sample included 39 479 adult respondents. Compared with ECTC-ineligible adults, ECTC-eligible adults were more often women (Table 1; 9137 [56.2%] vs 12 374 [49.6%]), married (10 005 [69.0%] vs 9833 [45.6%]), without medical conditions (8469 [57.5%] vs 9840 [43.4%]), employed or living with an employed person (13 795 [92.6%] vs 19 240 [82.2%]), and receiving SNAP benefits (3422 [23.9%] vs 2914 [11.9%]). They reported lower education levels (1792 [16.1%] reported less than a high school education vs 2106 [10.7%]) and household income to poverty ratios (2.1 vs 2.8), less frequent anxiety or depression (3419 [20.5%] vs 6608 [25.4%]), and were less often non-Hispanic White (7922 [48.7%] vs 15 782 [61.2%]). For the entire sample, compared with the pre-ECTC period, during the ECTC period, there were higher rates of anxiety and depression (1893 [25.4%] vs 8134 [22.9%]), receipt of SNAP benefits (1322 [19.7%] vs 5014 [16.6%]), insurance coverage (6070 [85.0%] vs 27 622 [82.7%]), and having a high school education or greater (6333 [89.2%] vs 29 248 [86.5%]).

**Table 1.** Sample Sociodemographic Characteristics by ECTC Eligibility and Policy Period

Characteristic	No. (%)			*P* value	No. (%)		*P* value
	Total	ECTC eligible	ECTC ineligible		Pre ECTC	During ECTC	
Total adults^a^	39 479	15 066 (43.1)	24 413 (56.9)	NA	32 460 (83.9)	7019 (16.1)	NA
Adult characteristics							
Age, mean (SD), y	41.0 (13.0)	37.3 (9.6)	43.9 (14.8)	<.001	41.0 (12.9)	41.1 (13.6)	.57
Participant sex							
Female	21 511 (52.4)	9137 (56.2)	12 374 (49.6)	<.001	17 613 (52.4)	3898 (52.9)	.50
Male	17 968 (47.6)	5929 (43.8)	12 039 (50.4)		14 847 (47.6)	3121 (47.1)	
Race and ethnicity							
Hispanic	7234 (21.7)	3814 (28.7)	3420 (16.4)	<.001	5822 (21.7)	1412 (22.2)	.61
Non-Hispanic American Indian/Alaska Native	321 (0.9)	128 (0.9)	193 (0.9)		267 (0.9)	54 (0.8)	
Non-Hispanic Asian	2205 (5.7)	933 (6.0)	1272 (5.4)		1768 (5.6)	437 (5.8)	
Non-Hispanic Black	5113 (13.7)	1918 (13.6)	3195 (13.8)		4234 (13.8)	879 (13.3)	
Non-Hispanic White	23 704 (55.8)	7922 (48.7)	15 782 (61.2)		19 641 (55.9)	4063 (55.5)	
Non-Hispanic multiracial/Other^b^	902 (2.2)	351 (2.1)	551 (2.2)		728 (2.1)	174 (2.5)	
Education level^c^							
Less than high school	3898 (13.0)	1792 (16.1)	2106 (10.7)	<.001	3212 (13.5)	686 (10.8)	<.001
High school/GED	11 363 (31.7)	4154 (30.8)	7209 (32.3)		9340 (31.4)	2023 (33.0)	
Some college	6931 (18.2)	2649 (17.4)	4282 (18.9)		5741 (18.5)	1190 (16.6)	
Associate degree	5625 (13.3)	2076 (12.8)	3549 (13.7)		4639 (13.5)	986 (12.4)	
Bachelor's degree	8000 (16.6)	2914 (15.3)	5086 (17.5)		6544 (16.1)	1456 (18.9)	
Master's degree	2980 (5.9)	1227 (6.3)	1753 (5.5)		2425 (5.7)	555 (6.8)	
Doctorate/professional degree	682 (1.4)	254 (1.3)	428 (1.4)		559 (1.3)	123 (1.5)	
Employment status^d^							
Employed	33 035 (86.7)	13 795 (92.6)	19 240 (82.2)	<.001	27 144 (86.6)	5891 (87.2)	.21
Unemployed	6444 (13.3)	1271 (7.4)	5173 (17.8)		5316 (13.4)	1128 (12.8)	
Marital status							
Married	19 838 (55.7)	10 005 (69.0)	9833 (45.6)	<.001	16 338 (55.7)	3500 (55.7)	.56
Living with a partner^e^	2673 (8.8)	1251 (10.3)	1422 (7.7)		2224 (8.9)	449 (8.8)	
Single	16 968 (35.5)	3810 (20.7)	13 158 (46.7)		13 898 (35.4)	3070 (35.5)	
Medical conditions							
None	18 309 (49.5)	8469 (57.5)	9840 (43.4)	<.001	15 108 (49.6)	3201 (48.7)	.63
1-3	18 486 (44.8)	6170 (39.8)	12 316 (48.6)		15 152 (44.)	3334 (45.4)	
3-6	2498 (5.4)	403 (2.6)	2095 (7.5)		2045 (5.4)	453 (5.5)	
≥7	186 (0.3)	24 (0.1)	162 (0.5)		155 (0.3)	31 (0.4)	
Anxiety or depression							
No	29 452 (76.7)	11 647 (79.5)	17 805 (74.6)	<.001	24 326 (77.1)	5126 (74.6)	<.001
Yes	10 027 (23.3)	3419 (20.5)	6608 (25.4)		8134 (22.9)	1893 (25.4)	
Health insurance							.003
Private HDHP^f^	8248 (19.4)	3094 (18.8)	5154 (19.8)	<.001	6806 (19.3)	1442 (19.6)	.003
Private traditional	16 326 (41.0)	5782 (37.5)	10 544 (43.6)		13 427 (40.8)	2899 (41.8)	
Medicaid	6604 (17.4)	3075 (20.7)	3529 (14.8)		5312 (17.2)	1292 (18.3)	
Other	2514 (5.4)	699 (3.9)	1815 (6.5)		2077 (5.4)	437 (5.3)	
Uninsured	5787 (16.9)	2416 (19.1)	3371 (15.3)		4838 (17.3)	949 (15.0)	
Urban/rural							
Large central metropolitan	11 438 (30.4)	4207 (29.8)	7231 (30.8)	.16	9331 (30.2)	2107 (31.2)	.77
Large fringe metropolitan	8126 (21.7)	3258 (22.5)	4868 (21.1)		6631 (21.8)	1495 (21.2)	
Medium/small metropolitan	13 386 (32.4)	5130 (32.2)	8256 (32.6)		11 087 (32.5)	2299 (32.1)	
Nonmetropolitan	6529 (15.4)	2471 (15.5)	4058 (15.4)		5411 (15.4)	1118 (15.5)	
Receipt of SNAP							
Yes	6336 (17.1)	3422 (23.9)	2914 (11.9)	<.001	5014 (16.6)	1322 (19.7)	<.001
No	33 143 (82.9)	11 644 (76.1)	21 499 (88.1)		27 446 (83.4)	5697 (80.3)	
Household characteristics							
Total children, mean (SD)	0.8 (1.0)	1.8 (0.7)	0	<.001	0.8 (1.0)	0.8 (1.0)	.27
Total adults, mean (SD)	2.1 (0.6)	2.3 (0.5)	2.1 (0.7)	<.001	2.1 (0.6)	2.1 (0.7)	.17
Income to poverty ratio, median (IQR)	2.5 (1.4-3.9)	2.1 (1.3-3.4)	2.8 (1.6-4.2)	<.001	2.5 (1.4-3.8)	2.5 (1.4-3.9)	.16

Abbreviations: ECTC, Expanded Child Tax Credit; GED, General Educational Development; HDHP, high deductible health plan; NA, not applicable; SNAP, Supplemental Nutrition Assistance Program.

^a^ Total weighted participants = 134 701 928. All percentages represent weighted values.

^b^ The National Health Interview Survey does not specify the racial and ethnic groups included in the category of “Other”. All race and ethnicity data were self-reported by survey respondents.

^c^ Educational level reflects highest education level attained in a participant’s household.

^d^ Represents household employment status. A household is considered employed if at least 1 adult works full time.

^e^ Individuals who reported living with a partner but who were unmarried were considered single when determining income eligibility for ECTC.

^f^ High-deductible health plans are defined as those with a personal deductible greater than $1400.

### ECTC and Overall Adult Health

In unadjusted analyses, following disbursement of monthly payments, the frequency of ECTC-eligible adults reporting excellent or very good health increased from 7633 (60.1%) to 1642 (63.1%). This was a 3.7 percentage point (pp) greater unadjusted increase than that experienced by ECTC-ineligible adults (Table 2). Among low-income households, ECTC-eligible adults reported a 1.2-pp greater unadjusted increase in excellent or very good health compared with ECTC-ineligible households. Among middle-income and upper-income households, ECTC-eligible adults reported a 4.1-pp greater unadjusted increase than ECTC-ineligible adults.

**Table 2.** Unadjusted Frequencies by ECTC Eligibility Group for Excellent or Very Good Health and Food Security Before vs During ECTC Monthly Payments With Difference-in-Differences Results^a^^,^^b^^,^^c^^,^^d^

Characteristic	Frequency, ECTC-eligible adults			Frequency, ECTC-ineligible adults			Difference-in-differences, pp
	Pre ECTC, No. (%)	During ECTC, No. (%)	Difference, pp	Pre ECTC, No. (%)	During ECTC, No. (%)	Difference, pp	
**Excellent or very good health**							
All participants	7633 (60.1)	1642 (63.1)	+2.9	10 778 (54.9)	2309 (54.1)	−0.7	+3.7
≤200% FPL^e^	2908 (51.3)	650 (55.6)	+4.4	2510 (43.9)	593 (47.5)	+3.6	+1.2
>200% FPL	4725 (68.4)	992 (69.7)	+1.3	8268 (60.2)	1716 (57.4)	−2.8	+4.1
**Food secure**							
All participants	10 950 (87.8)	2421 (91.4)	+3.7	17 839 (89.1)	3953 (91.2)	+2.0	+1.7
≤200% FPL^f^	4427 (78.9)	1021 (85.0)	+6.1	4857 (76.9)	1093 (79.1)	+2.2	+3.8
>200% FPL^g^	6523 (96.0)	1400 (97.1)	+1.1	12 982 (95.0)	2860 (97.0)	+1.9	−0.8

Abbreviations: ECTC, Expanded Child Tax Credit; FPL, federal poverty line; pp, percentage point.

^a^ Difference-in-differences estimates were calculated by subtracting the during-ECTC vs pre-ECTC frequency difference for ECTC-ineligible households from that for ECTC-eligible households.

^b^ Overall health status and food security are dichotomous variables. Those who did not answer excellent or very good health reported good, fair, or poor health. Those who did not report food security reported food insecurity. The frequency of good, fair, or poor health and food insecurity can be determined by subtracting the frequency in the table from 1. The percentage change and difference-in-differences estimates can be determined by flipping the sign on the differences shown in the table.

^c^ All adults: n = 39 479; low-income adults: n = 14 670; middle-income and upper-income adults: n = 24 809.

^d^ All percentages represent weighted values.

^e^ Federal poverty line determined from 2020 Census Bureau thresholds (eTable 1 in Supplement 1).

^f^ Low-income households.

^g^ Middle-income and upper-income households.

In adjusted difference-in-differences analyses, ECTC eligibility was associated with an increase in excellent and very good health (Table 3 and Figure, A; difference in differences, +3.0 pp; 95% CI, 0.2-5.7). In income-stratified analyses, this association was significant for middle-income and upper-income adults (difference-in-differences, +3.7 pp; 95% CI, 0.5-6.9). For low-income households, ECTC-eligible adults and ECTC-ineligible adults reported increases in excellent and very good health (+4.0 pp [95% CI, 0.4-7.5] vs +3.5 pp [95% CI, 0.3-6.6], respectively) resulting in a difference-in-differences estimate of +0.5 pp (95% CI, −4.3 to 5.2). Controlling for food security had a negligible attenuating association with the difference-in-differences estimator (eTable 2 in Supplement 1), failing to support food security as a mediator of changes in adult health.

**Table 3.** Predicted Probabilities by ECTC Eligibility Group for Excellent or Very Good Health and Food Security Before vs During ECTC Monthly Payments With Diff-in-Diff Results^a^^,^^b^^,^^c^^,^^d^

Characteristic	Predicted probability, ECTC-eligible adults			Predicted probability, ECTC-ineligible adults			Diff-in-diff	
	Pre ECTC % (95% CI)	During ECTC % (95% CI)	Difference, pp (95% CI)	Pre ECTC % (95% CI)	During ECTC % (95% CI)	Difference, pp (95% CI)	Difference, pp (95% CI)	*P* value
**Excellent or very good health**								
All participants	56.4 (55.3 to 57.5)	59.1 (57.1 to 61.1)	+2.7 (0.5 to 4.9)	57.6 (56.7 to 58.5)	57.4 (55.8 to 59.0)	−0.3 (−1.9 to 1.5)	+3.0 (0.2 to 5.7)	.03
≤200% FPL^e^^,^^f^	53.1 (51.4 to 54.8)	57.1 (53.8 to 60.3)	+4.0 (0.4 to 7.5)	52.7 (51.0 to 54.4)	56.2 (53.2 to 59.2)	+3.5 (0.3 to 6.6)	+0.5 (−4.3 to 5.2)	.84
>200% FPL^g^	58.3 (56.7 to 59.8)	59.9 (57.5 to 62.3)	+1.6 (−1.0 to 4.2)	58.9 (57.7 to 60.1)	56.8 (54.9 to 58.8)	−2.1 (−4.1 to −0.1)	+3.7 (0.5 to 6.9)	.02
**Food secure**								
All participants	87.9 (87.2 to 88.7)	91.9 (90.6 to 93.2)	+4.0 (2.6 to 5.4)	89.0 (88.2 to 89.7)	91.1 (90.0 to 92.1)	+2.1 (0.9 to 3.3)	+1.9 (0.1 to 3.7)	.04
≤200% FPL	84.5 (83.1 to 85.9)	90.8 (88.2 to 93.5)	+6.3 (3.6 to 9.0)	83.3 (81.6 to 85.0)	85.7 (83.0 to 88.3)	+2.4 (−0.5 to 5.9)	+3.9 (0.0 to 7.9)	.05
>200% FPL	92.6 (91.8 to 93.5)	94.3 (93.3 to 95.3)	+1.7 (0.5 to 2.8)	92.7 (91.8 to 93.5)	94.7 (93.8 to 95.6)	+2.0 (1.1 to 2.8)	−0.3 (−1.7 to 1.1)	.66

Abbreviations: diff-in-diff, difference in differences; ECTC, Expanded Child Tax Credit; FPL, federal poverty line; pp, percentage point.

^a^ Models adjusted for age, sex, race and ethnicity, highest household educational attainment, household employment status, chronic disease burden, anxiety or depression diagnosis, health insurance, number of adults, rurality, and receipt of Supplemental Nutrition Assistance Program benefits.

^b^ Overall health status and food security are dichotomous variables. Those who did not answer excellent or very good health reported good, fair, or poor health. Those who did not report food security reported food insecurity. The probability of good, fair, or poor health and food insecurity can be determined by subtracting the probability in the table from 1. The percentage change and diff-in-diff estimates can be determined by flipping the sign on the differences in the table.

^c^ Diff-in-diff estimates were calculated by subtracting the adjusted during-ECTC vs pre-ECTC probability difference for ECTC-ineligible households from that for ECTC-eligible households.

^d^ All adults: n = 39 479; low-income adults: n = 14 670; middle-income and upper-income adults: n = 24 809.

^e^ Federal poverty line determined from 2020 Census Bureau thresholds (eTable 1 in Supplement 1).

^f^ Low-income households.

^g^ Middle-income and upper-income households.

**Figure.**
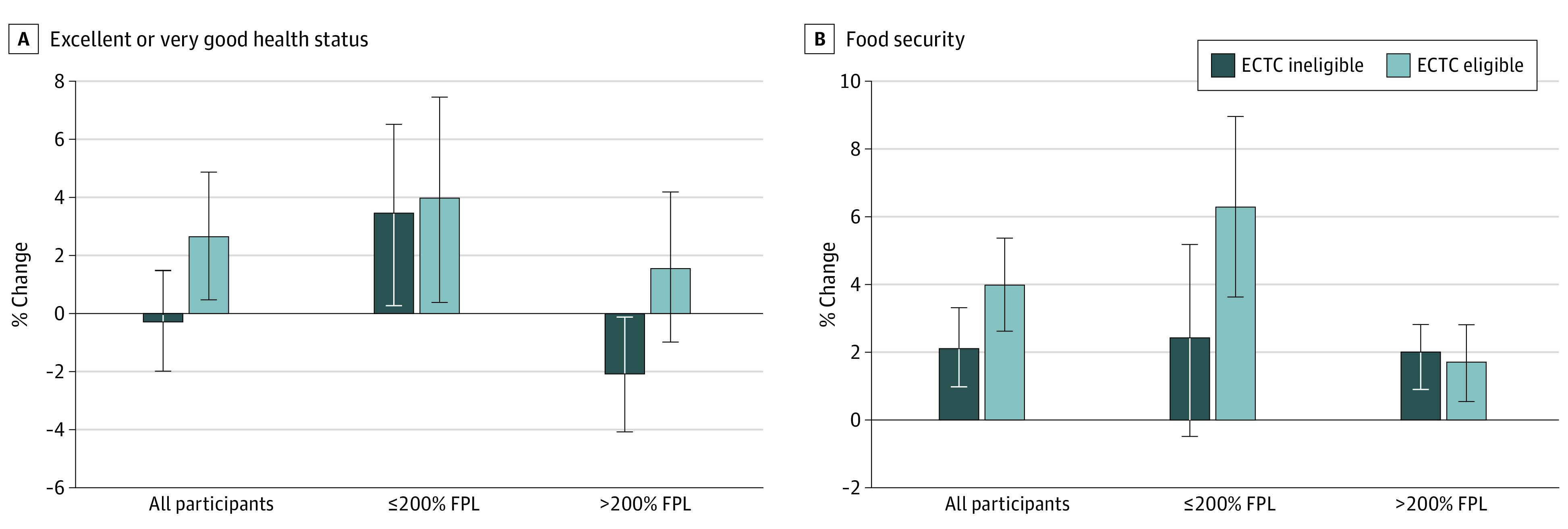
Adjusted Marginal Change in Excellent or Very Good Health and Food Security Following Disbursement of Expanded Child Tax Credit (ECTC) Monthly Payments by Eligibility Group Models adjust for age, sex, race and ethnicity, highest household educational attainment, household employment status, chronic disease burden, chronic mental health condition, health insurance, number of adults, rurality, receipt of Supplemental Nutrition Assistance Program benefits. Marginal change is calculated by subtracting the adjusted pre-ECTC probability from the during-ECTC probability. Error bars represent 95% CIs. All adults: n = 39 479; low-income adults (≤200% federal poverty line [FPL]): n = 14 670; middle-income and upper-income adults (>200% FPL): n = 24 809. Federal poverty line (FPL) determined from 2020 Census Bureau thresholds (eTable 1 in the Supplement).

### ECTC and Household Food Security

In unadjusted analyses, following disbursement of monthly payments, ECTC-eligible households reported an increase in food security from 10 950 (87.8%) to 2421 (91.4%), a 1.7-pp greater unadjusted increase than ECTC-ineligible households (Table 2). In income-stratified analyses, low-income ECTC-eligible households reported a 3.8-pp greater unadjusted increase in food security than ECTC-ineligible households. Conversely, middle-income and upper-income ECTC-ineligible households reported a 0.8-pp greater unadjusted increase in food security than ECTC-eligible households.

In adjusted difference-in-differences analyses, ECTC eligibility was associated with an increase in overall food security (Table 3 and Figure, B; difference-in-differences, +1.9 pp; 95% CI, 0.1-3.7). In income-stratified analyses, this association was significant for low-income households, with ECTC-eligible adults reporting a larger increase in food security than ECTC-ineligible adults (+6.3 pp [95% CI, 3.6-9.0] vs +2.4 pp [95% CI, −0.5 to 5.9]; difference-in-differences, +3.9 pp; 95% CI, 0-7.9).

### Sensitivity Analyses

Increasing the income eligibility threshold from $75 000 to $112 500 increased the total sample size to 44 096 households. Using this sample did not substantially change difference-in-differences estimates (eTable 3 in Supplement 1). Logistic and ordinal logistic regression models produced similar associations between ECTC eligibility and overall health and a slightly more attenuated association between ECTC eligibility and overall food security (eTables 4 and 5 in Supplement 1). Marginal probabilities and difference-in-differences estimates using the original scales for overall health and food security are provided in eTable 9 in Supplement 1. Analyses that excluded participants interviewed in 2019 did not substantially change the overall association between ECTC eligibility and health and food security (eTable 6 in Supplement 1). Excluding receipt of SNAP as a covariate did not substantially alter the association between ECTC eligibility and overall health or food security (eTable 7 in Supplement 1).

## Discussion

In this cross-sectional analysis of nationally representative survey data, ECTC eligibility was associated with improved adult health and household food security. To our knowledge, this study is the first to show a significant association between ECTC eligibility and improved overall health. To date, only 2 published studies have assessed this topic, with 1 finding an association between the ECTC and a reduction in depression and anxiety symptoms among low-income adults and the other finding no association between ECTC monthly payments and changes in life satisfaction or mental health.^18,19^ While these studies focused on important measures of mental health, the present study used overall self-reported health, a well-validated measure that has been shown to predict health outcomes, including mortality.^36,37^

We hypothesize that the positive association between ECTC eligibility and adult overall health was likely mediated through associated reductions in poverty and financial hardship.^13^ Ample research supports this association, with reductions in poverty associated with reduced mortality, disability, heart disease, and diabetes.^2,3,4,38^ Additionally, the study findings are consistent with prior research on cash transfer programs, such as the Earned Income Tax Credit, which has been associated with improvements in maternal health, infant birth weight, and self-reported well-being.^39,40,41,42,43^ While studies have shown a positive association between food security and health,^44^ we did not find compelling evidence that improvements in health were mediated by improved food security.

To our knowledge, the present study is also the first to use nationally representative data to show improvements in food security associated with ECTC eligibility. While prior studies using nationally representative data reported on changes in food insufficiency (a single-question measure), this study measured food security, a more descriptive 4-level construct validated by the USDA.^30^ The study findings were consistent with prior research on this topic and potentially provide additional evidence of the positive association between income and food security.^20,21,22,23,45,46^

The association of ECTC eligibility with health was most pronounced in middle-income and upper-income households and not low-income households. We hypothesize several reasons for this surprising finding. First, it is possible that the additional income provided by the ECTC may have been insufficient to reverse the negative health effects of long-standing economic hardship.^47,48,49,50,51^ The COVID-19 pandemic exacerbated many socioeconomic and racial health disparities, disproportionately affecting low-income Americans compared with other income groups.^52,53,54^ Given the unprecedented and disproportionate level of health-related stress experienced by low-income adults during the study period, the ECTC may have been insufficient to change their overall health trajectory or buffer all the increased health threats they faced. Second, it is estimated that fewer than half of eligible low-income families received monthly payments.^55,56^ Given that this study assessed ECTC eligibility and not receipt, this study may have been underpowered to show changes in health for low-income adults who actually received the payments. Third, our model used difference-in-differences analyses; therefore, improvements in health experienced by the control group, low-income ECTC-ineligible adults, diminished the magnitude of the positive association between ECTC eligibility and health. We hypothesize that the expansion of COVID19–related social support programs, including unemployment, SNAP benefits, economic impact payments, and health insurance expansions, may have had a larger association with outcomes for ECTC-ineligible households given that they are inherently comprised of only working-age adults who would have each been eligible to benefit from these programs.^12,32,57,58^ While we attempted to control for these additional benefits, particularly receipt of SNAP and changes in health insurance, these policies may have disproportionately affected low-income ineligible adults, thus attenuating our ability to measure the ECTC’s association with health in low-income households.

### Policy Implications

While other countries have adopted generous permanent monthly cash transfer programs for families, such as the Canada Child Benefit, the US has favored cash transfer programs tied to employment and tax refunds, such as the Child Tax Credit and Earned Income Tax Credit.^16,39,59^ The ECTC temporarily increased the generosity of the benefit to more closely reflect that of other high-income countries and represents the US government’s largest recent attempt at an unconditional cash transfer program for families with children.^6^ This study provides evidence that this program may have improved adult health and household food security. As future antipoverty measures are being designed, unconditional cash transfer programs like the ECTC should be considered as a potentially effective method to improve population health. Furthermore, in light of research indicating that many low-income families failed to receive monthly payments, future investigation should focus on the implementation of cash transfer programs to ensure the equitable disbursement of future payments.^55,56^ Physicians and allied health professionals can serve a critical role in improving the likelihood of patients receiving these benefits.^60^

### Limitations

This study was conducted during the COVID-19 pandemic; thus, it may have been subject to unmeasured confounding from associated job losses, changes in health, and expansions to social programs, including unemployment and SNAP benefits.^32,58^ We attempted to address this by using a difference-in-differences model that used childless households as a control to account for temporal changes associated with the pandemic. Additionally, given that the NHIS did not specifically report receipt of the ECTC, we derived eligibility from income and family composition, which likely did not perfectly reflect receipt of the ECTC. As such, the analysis represents an intent-to-treat approach, which might underestimate the magnitude of associations for those who received the ECTC. In addition, the NHIS does not uniformly provide the relationship of the sample adult to the household child/children. Because of this, the findings of this study cannot speak to the ECTC’s effect on a specific individual within a household unit (eg, as a parent or adult sibling) but instead evaluates the effect of ECTC eligibility on any adult living in a household with a child. Lastly, the dichotomization of ordinal variables can be followed by a loss of information.^61^ Fortunately, in sensitivity analyses, the models using binary outcomes demonstrated consistency with those using the ordinal specification.

## Conclusions

In this cross-sectional study using nationally representative survey data, we found that ECTC eligibility was associated with improved self-reported overall health and improved household food security. The study findings potentially provide evidence that unrestricted monthly cash transfer programs like the ECTC are not only effective antipoverty measures but also may be powerful population health tools. Future studies should continue to examine this policy’s association with health, barriers to its receipt, and its cost-effectiveness.

## Notes

Supplement 1. **eTable 1. **2020 Census Bureau poverty thresholds **eTable 2.** Evaluation of food security as a mediator for the association between ECTC eligibility and overall adult health **eTable 3.** Adjusted difference-in-differences results with alternative ECTC eligibility threshold **eTable 4. **Adjusted difference-in-differences results with logistic regression models **eTable 5. **Adjusted difference-in-differences results with ordinal logistic regression model **eTable 6. **Adjusted difference-in-differences results when excluding 2019 **eTable 7.** Adjusted difference-in-differences results without adjustment for receipt of SNAP **eTable 8. **Linear probability model comparing changes in adult overall health and food security by ECTC eligibility **eTable 9.** Unadjusted frequencies by ECTC eligibility group for overall health status and food security states before and during ECTC monthly payments with difference-in-differences results **eFigure 1. **Frequency of adults reporting excellent or very good health, National Health Interview Survey, January 2019 to December 2021 **eFigure 1B.** Frequency of adults reporting food security, National Health Interview Survey, January 2019 to December 2021

Supplement 2. Data sharing statement
