# Supplementary material for: Changes in Self-Reported Adult Health and Household Food Security With the 2021 Expanded Child Tax Credit Monthly Payments
Source: JAMA Health Forum. 2023 Jun 24;4(6):e231672. doi: 10.1001/jamahealthforum.2023.1672 (PMC10290752; doi:10.1001/jamahealthforum.2023.1672)
Supplement: Supplement 1. — eTable 1. 2020 Census Bureau poverty thresholds eTable 2. Evaluation of food security as a mediator for the association between ECTC eligibility and overall adult health eTable 3. Adjusted difference-in-differences results with alternative ECTC eligibility threshold eTable 4. Adjusted difference-in-differences results with logistic regression models eTable 5. Adjusted difference-in-differences results with ordinal logistic regression model eTable 6. Adjusted difference-in-differences results when excluding 2019 eTable 7. Adjusted difference-in-differences results without adjustment for receipt of SNAP eTable 8. Linear probability model comparing changes in adult overall health and food security by ECTC eligibility eTable 9. Unadjusted frequencies by ECTC eligibility group for overall health status and food security states before and during ECTC monthly payments with difference-in-differences results eFigure 1. Frequency of adults reporting excellent or very good health, National Health Interview Survey, January 2019 to December 2021 eFigure 1B. Frequency of adults reporting food security, National Health Interview Survey, January 2019 to December 2021 [file jamahealthforum-e231672-s001.pdf]

Supplemental Online Content

Rook JM, Yama CL, Schickedanz AB, Feuerbach AM, Lee SL, Wisk LE. Changes in self-reported adult health and household food security with the 2021 Expanded Child Tax Credit monthly payments. *JAMA Health Forum*. 2023;4(6):e231672. doi:10.1001/jamahealthforum.2023.1672

eTable 1. 2020 Census Bureau poverty thresholds

eTable 2. Evaluation of food security as a mediator for the association between ECTC eligibility and overall adult health

eTable 3. Adjusted difference-in-differences results with alternative ECTC eligibility threshold

eTable 4. Adjusted difference-in-differences results with logistic regression models

eTable 5. Adjusted difference-in-differences results with ordinal logistic regression model

eTable 6. Adjusted difference-in-differences results when excluding 2019

eTable 7. Adjusted difference-in-differences results without adjustment for receipt of SNAP

eTable 8. Linear probability model comparing changes in adult overall health and food security by ECTC eligibility

eTable 9. Unadjusted frequencies by ECTC eligibility group for overall health status and food security states before and during ECTC monthly payments with difference-in-differences results

eFigure 1. Frequency of adults reporting excellent or very good health, National Health Interview Survey, January 2019 to December 2021

eFigure 1B. Frequency of adults reporting food security, National Health Interview Survey, January 2019 to December 2021

This supplemental material has been provided by the authors to give readers additional information about their work.

© 2023 Rook JM et al. *JAMA Health Forum.*

eTable 1: 2020 Census Bureau poverty thresholds

**Household Size (people)** [a]	**Federal Poverty Line (dollars)**
1	$14,000.00
2	$18,000.00
3	$21,000.00
4	$27,000.00
5	$32,000.00
6+	$36,000.00

[a] The National Health Interview Survey top codes family size at 6 people.

© 2023 Rook JM et al. *JAMA Health Forum.*

eTable 2: Evaluation of food security as a mediator for the association between ECTC eligibility and overall adult health [a-c]

	Standard Model			Adjusting for Food Security		
	Coeff	95% CI		Coeff	95% CI	
**All Households**						
**Eligible vs Not**	-0.012	-0.027	0.002	-0.011	-0.026	0.003
**During vs Pre**	-0.003	-0.020	0.015	-0.004	-0.022	0.013
**Diff-in-Diff**	0.030	0.002	0.060	0.028	0.001	0.055
**Low-Income Households (≤200% FPL)[d]**						
**Eligible vs Not**	0.004	-0.020	0.028	0.003	-0.021	0.027
**During vs Pre**	0.035	0.003	0.066	0.033	0.002	0.065
**Diff-in-Diff**	0.005	-0.043	0.052	0.002	-0.045	0.049
**Middle- and Upper-Income Households (>200% FPL)**						
**Eligible vs Not**	-0.006	-0.024	0.012	-0.006	-0.024	0.012
**During vs Pre**	-0.021	-0.041	-0.001	-0.023	-0.044	-0.003
**Diff-in-Diff**	0.037	0.005	0.069	0.037	0.005	0.069

[a] Linear probability models adjust for age, sex, race/ethnicity, highest household educational attainment, household employment status, chronic disease burden, anxiety or depression diagnosis, health insurance, number of adults, rurality, receipt of SNAP benefits. [b] All adults: N=39,479; low-income adults: N=14,670; middle- and upper-income adults: N=24,809.[c] Excellent or very good health is being compared to good, fair, or poor health. [d] Federal Poverty Line determined from 2020 Census Bureau thresholds (eTable 1).

Abbreviations: ECTC – Expanded Child Tax Credit; Coeff – Coefficient; Eligible vs Not – ECTC-Eligible versus ECTC-Ineligible; During vs Pre – During-ECTC versus Pre-ECTC; Diff-in-Diff – Difference-in-Differences; FPL – Federal Poverty Line

© 2023 Rook JM et al. *JAMA Health Forum.*

eTable 3: Adjusted difference-in-differences results with alternative ECTC eligibility threshold [a-c]

	Excellent or Very Good Health[d]			Food Security[e]		
	Coeff	95% CI		Coeff	95% CI	
**All Households**						
**Eligible vs Not**	-0.008	-0.021	0.006	-0.010	-0.020	-0.001
**During vs Pre**	-0.001	-0.016	0.016	0.017	0.007	0.028
**Diff-in-Diff**	**0.025**	**-0.001**	**0.051**	**0.023**	**0.007**	**0.041**
**Low-Income Households (≤200% FPL) [f]**						
**Eligible vs Not**	0.003	-0.020	0.028	0.012	-0.012	0.036
**During vs Pre**	0.035	0.003	0.066	0.024	-0.005	0.052
**Diff-in-Diff**	**0.005**	**-0.043**	**0.053**	**0.039**	**0.000**	**0.079**
**Middle- and Upper-Income Households (>200% FPL)**						
**Eligible vs Not**	0.001	-0.015	0.018	0.000	-0.007	0.007
**During vs Pre**	-0.015	-0.033	0.004	0.015	0.007	0.022
**Diff-in-Diff**	**0.027**	**-0.003**	**-0.057**	**0.004**	**-0.009**	**0.017**

[a] Linear probability models adjust for age, sex, race/ethnicity, highest household educational attainment, household employment status, chronic disease burden, anxiety or depression diagnosis, health insurance, number of adults, rurality, receipt of SNAP benefits. [b] All adults: N=44,096; low-income adults: N=14,670; middle- and upper-income adults: N=29,426. [c] Model Includes single adults with household incomes less than $112,500 instead of standard threshold of $75,000. [d] Excellent or very good health compared to good, fair, or poor health. [e] Food security compared to food insecurity. [f] Federal Poverty Line determined from 2020 Census Bureau thresholds (eTable 1).

Abbreviations: ECTC – Expanded Child Tax Credit; Coeff – Coefficient; Eligible vs Not – ECTC-Eligible versus ECTC-Ineligible; During vs Pre – During-ECTC versus Pre-ECTC; Diff-in-Diff – Difference-in-Differences; FPL – Federal Poverty Line

© 2023 Rook JM et al. *JAMA Health Forum.*

eTable 4: Adjusted difference-in-differences results with logistic regression models [a,b]

	Excellent or Very Good Health[c]			Food Security[d]		
	OR	95% CI		OR	95% CI	
**All Households**						
**Eligible vs Not**	0.93	0.87	1.00	0.92	0.81	1.04
**During vs Pre**	0.98	0.90	1.08	1.30	1.11	1.53
**Diff-in-Diff**	1.17	1.01	1.34	1.21	0.95	1.55
**Low-Income Households (≤200% FPL)[e]**						
**Eligible vs Not**	1.03	0.91	1.17	1.08	0.93	1.26
**During vs Pre**	1.22	1.02	1.45	1.15	0.95	1.38
**Diff-in-Diff**	1.00	0.78	1.28	1.37	1.03	1.82
**Middle- and Upper-Income Households (>200% FPL)**						
**Eligible vs Not**	0.96	0.87	1.05	0.97	0.79	1.19
**During vs Pre**	0.90	0.81	1.00	1.77	1.35	2.31
**Diff-in-Diff**	1.21	1.02	1.44	0.89	0.55	1.42

[a] Models adjust for age, sex, race/ethnicity, highest household educational attainment, household employment status, chronic disease burden, anxiety or depression diagnosis, health insurance, number of adults, rurality, receipt of SNAP benefits. [b] All adults: N=39,479; low-income adults: N=14,670; middle- and upper-income adults: N=24,809. [c] Excellent or very good health compared to good, fair, or poor health. [d] Food security compared to food insecurity. [e] Federal Poverty Line determined from 2020 Census Bureau thresholds (eTable 1).

Abbreviations: ECTC – Expanded Child Tax Credit; OR – Odds Ratio; Eligible vs Not – ECTC-Eligible versus ECTC-Ineligible; During vs Pre – During-ECTC versus Pre-ECTC; Diff-in-Diff – Difference-in-Differences; FPL – Federal Poverty Line

© 2023 Rook JM et al. *JAMA Health Forum.*

eTable 5: Adjusted difference-in-differences results with ordinal logistic regression model [a-d]

	Improved Health			Improved Food Security		
	OR	95% CI		OR	95% CI	
**All Households**						
**Eligible vs Not**	0.94	0.88	0.99	0.87	0.79	0.95
**During vs Pre**	0.99	0.92	1.07	1.24	1.10	1.41
**Diff-in-Diff**	1.14	1.01	1.29	1.15	0.96	1.38
**Low-Income Households (≤200% FPL)[e]**						
**Eligible vs Not**	1.02	0.92	1.13	1.01	0.89	1.15
**During vs Pre**	1.14	0.99	1.32	1.16	0.99	1.37
**Diff-in-Diff**	1.03	0.83	1.26	1.29	1.02	1.63
**Middle- and Upper-Income Households (>200% FPL)**						
**Eligible vs Not**	0.97	0.90	1.05	0.94	0.81	1.10
**During vs Pre**	0.92	0.84	1.00	1.39	1.15	1.68
**Diff-in-Diff**	1.18	1.02	1.36	0.87	0.64	1.19

[a] Models adjust for age, sex, race/ethnicity, highest household educational attainment, household employment status, chronic disease burden, anxiety or depression diagnosis, health insurance, number of adults, rurality, receipt of SNAP benefits. [b] All adults: N=39,479; low-income adults: N=14,670; middle- and upper-income adults: N=24,809. [c] The Brant Test demonstrated no violation of the proportional odds assumption. [d] Overall health is evaluated on a five-level scale (excellent, very good, good, fair, and poor). Food security is evaluated on a four-level scale (high food security, marginal food security, low food security, very low food security). [e] Federal Poverty Line determined from 2020 Census Bureau thresholds (eTable 1).

Abbreviations: ECTC – Expanded Child Tax Credit; OR – Odds Ratio; Eligible vs Not – ECTC-Eligible versus ECTC-Ineligible; During vs Pre – During-ECTC versus Pre-ECTC; Diff-in-Diff – Difference-in-Differences; FPL – Federal Poverty Line

© 2023 Rook JM et al. *JAMA Health Forum.*

eTable 6: Adjusted difference-in-differences results when excluding 2019 [a,b]

	Excellent or Very Good Health[c]			Food Security[d]		
	Coeff	95% CI		Coeff	95% CI	
**All Households**						
**Eligible vs Not**	-0.009	-0.027	0.008	-0.016	-0.028	-0.003
**During vs Pre**	-0.009	-0.266	0.009	0.010	-0.002	0.022
**Diff-in-Diff**	**0.027**	**-0.002**	**0.056**	**0.021**	**0.002**	**0.039**
**Low-Income Households (≤200% FPL)[e]**						
**Eligible vs Not**	0.015	-0.014	0.044	-0.001	-0.030	0.027
**During vs Pre**	0.029	-0.004	0.062	0.005	-0.024	0.035
**Diff-in-Diff**	**-0.005**	**-0.055**	**0.045**	**0.047**	**0.007**	**0.088**
**Middle- and Upper-Income Households (>200% FPL)**						
**Eligible vs Not**	-0.009	-0.031	0.013	0.001	-0.008	0.009
**During vs Pre**	-0.026	-0.047	-0.005	0.014	0.005	0.022
**Diff-in-Diff**	**0.040**	**0.006**	**0.074**	**-0.006**	**-0.021**	**0.009**

[a] Linear probability models adjust for age, sex, race/ethnicity, highest household educational attainment, household employment status, chronic disease burden, anxiety or depression diagnosis, health insurance, number of adults, rurality, receipt of SNAP benefits. [b] All adults: N=27,837; low-income adults: N=10,067; middle- and upper-income adults: N=17,770. [c] Excellent or very good health compared to good, fair, or poor health. [d] Food security compared to food insecurity. [e] Federal Poverty Line determined from 2020 Census Bureau thresholds (eTable 1).

Abbreviations: ECTC – Expanded Child Tax Credit; Coeff – Coefficient; Eligible vs Not – ECTC-Eligible versus ECTC-Ineligible; During vs Pre – During-ECTC versus Pre-ECTC; Diff-in-Diff – Difference-in-Differences; FPL – Federal Poverty Line

© 2023 Rook JM et al. *JAMA Health Forum.*

eTable 7: Adjusted difference-in-differences results without adjustment for receipt of SNAP [a,b]

	Excellent or Very Good Health[c]			Food Security[d]		
	**Coeff**	**95% CI**		**Coeff**	**95% CI**	
	**All Households**					
**Eligible vs Not**	-0.020	-0.034	-0.006	-0.026	-0.036	-0.016
**During vs Pre**	-0.003	-0.021	0.014	0.019	0.008	0.031
**Diff-in-Diff**	**0.027**	**0.000**	**0.055**	**0.014**	**-0.004**	**0.032**
	**Low-Income Households (≤200% FPL)[e]**					
**Eligible vs Not**	-0.005	-0.028	0.019	-0.005	-0.028	0.018
**During vs Pre**	0.033	0.001	0.065	0.020	-0.008	0.049
**Diff-in-Diff**	**0.003**	**-0.044**	**0.051**	**0.036**	**-0.003**	**0.075**
	**Middle- and Upper-Income Households (>200% FPL)**					
**Eligible vs Not**	-0.009	-0.027	0.009	-0.005	-0.013	0.003
**During vs Pre**	-0.021	-0.042	-0.001	0.019	0.011	0.027
**Diff-in-Diff**	**0.034**	**0.002**	**0.066**	**-0.007**	**-0.021**	**0.006**

[a] Linear probability models adjust for age, sex, race/ethnicity, highest household educational attainment, household employment status, chronic disease burden, anxiety or depression diagnosis, health insurance, number of adults, and rurality. [b] All adults: N=39,479; low-income adults: N=14,670; middle- and upper-income adults: N=24,809. . [c] Excellent or very good health compared to good, fair, or poor health. [d] Food security compared to food insecurity. [e] Federal Poverty Line determined from 2020 Census Bureau thresholds (eTable 1).

Abbreviations: SNAP – Supplemental Nutrition Assistance Program; ECTC – Expanded Child Tax Credit; Coeff – Coefficient; Eligible vs Not – ECTC-Eligible versus ECTC-Ineligible; During vs Pre – During-ECTC versus Pre-ECTC; Diff-in-Diff – Difference-in-Differences; FPL – Federal Poverty Line

© 2023 Rook JM et al. *JAMA Health Forum.*

eTable 8: Linear probability model comparing changes in adult overall health and food security by ECTC eligibility [a,b]

	Excellent or Very Good Health[c]			Food Security[d]		
	Coeff	95% CI		Coeff	95% CI	
**All Households**						
**Eligible vs Not**	-0.012	-0.027	0.002	-0.010	-0.021	0.000
**During vs Pre**	-0.003	-0.020	0.015	0.021	0.010	0.033
**Diff-in-Diff**	0.030	0.002	0.057	0.019	0.001	0.037
**Low-Income Households (≤200% FPL)[e]**						
**Eligible vs Not**	0.004	-0.020	0.028	0.012	-0.012	0.036
**During vs Pre**	0.035	0.003	0.066	0.024	-0.005	0.052
**Diff-in-Diff**	0.005	-0.043	0.052	0.039	0.000	0.079
**Middle- and Upper-Income Households (>200% FPL)**						
**Eligible vs Not**	-0.006	-0.024	0.012	-0.001	-0.009	0.007
**During vs Pre**	-0.021	-0.041	-0.001	0.020	0.011	0.028
**Diff-in-Diff**	0.037	0.005	0.069	-0.003	-0.017	0.011

[a] Linear probability models adjust for age, sex, race/ethnicity, highest household educational attainment, household employment status, chronic disease burden, anxiety or depression diagnosis, health insurance, number of adults, rurality, receipt of SNAP benefits. [b] All adults: N=39,479; low-income adults: N=14,670; middle- and upper-income adults: N=24,809. [c] Excellent or very good health compared to good, fair, or poor health. [d] Food security compared to food insecurity. [e] Federal Poverty Line determined from 2020 Census Bureau thresholds (eTable 1).

Abbreviations: ECTC – Expanded Child Tax Credit; Coeff – Coefficient; Eligible vs Not – ECTC-Eligible versus ECTC-Ineligible; During vs Pre – During-ECTC versus Pre-ECTC; Diff-in-Diff – Difference-in-Differences; FPL – Federal Poverty Line

© 2023 Rook JM et al. *JAMA Health Forum.*

eTable 9: Unadjusted frequencies by ECTC eligibility group for overall health status and food security states before and during ECTC monthly payments with difference-in-differences results [a-c]

	Frequency, ECTC-Eligible Adults (%)			Frequency, ECTC-Ineligible Adults (%)			Diff-in-diff	
Characteristic	Pre-ECTC	During-ECTC	Difference (pp)	Pre-ECTC	During-ECTC	Difference (pp)	Unadjusted Difference	Adjusted Difference (pp, 95% CI)
**Excellent health**								
All Participants	26.4	28.1	+1.6	22.6	21.4	-1.2	+2.8	+2.0 (0.1 to 4.0)
≤ 200% FPL [d]	23.0	25.1	+2.1	20.3	20.8	+0.5	+1.6	+0.5 (-3.0 to 3.9)
> 200% FPL	29.6	30.7	+1.1	23.7	21.7	-2.0	+3.1	+2.5 (0.3 to 4.7)
**Very Good Health**								
All Participants	33.7	35.0	+1.3	32.3	32.7	+0.4	+0.9	+1.1 (0.1 to 2.2)
≤ 200% FPL	28.2	30.5	+2.3	23.6	26.7	+3.1	-0.8	+0.2 (-1.5 to 1.9)
> 200% FPL	38.8	39.0	+0.2	36.4	35.6	-0.8	+1.0	+1.5 (0.2 to 2.7)
**Good Health**								
All Participants	28.0	26.5	-1.5	29.0	29.9	+0.9	-2.4	-2.0 (-3.9 to -0.1)
≤ 200% FPL [d]	31.5	29.5	-2.0	29.7	28.2	-1.5	-0.5	-0.4 (-3.3 to 2.6)
> 200% FPL	24.8	23.9	-0.9	28.6	30.7	+2.1	-3.0	-2.7 (-5.1 to -0.3)
**Fair Health**								
All Participants	9.9	8.4	-1.5	12.3	11.8	-0.4	-1.1	-1.0 (-1.9 to -0.1)
≤ 200% FPL [d]	14.2	11.6	-2.6	18.8	17.1	-1.7	-1.1	-0.2 (-2.0 to 1.6)
> 200% FPL	5.9	5.6	-0.3	9.2	9.3	+0.1	-0.4	-1.1 (-2.0 to -0.1)
**Poor Health**								
All Participants	1.9	1.9	0.0	3.9	4.1	+0.2	-0.2	-0.2 (-0.4 to 0.0)
≤ 200% FPL [d]	3.0	3.2	+0.2	7.7	7.2	-0.5	+0.7	-0.0 (-0.4 to 0.3)
> 200% FPL	0.9	0.8	-0.1	2.1	2.7	+0.6	-0.7	-0.2 (-0.4 to -0.0)
**High Food Security**								
All Participants	78.4	82.7	+4.3	82.0	84.5	+2.5	+1.8	+1.9 (-0.2 to 4.0)
≤ 200% FPL	64.5	72.3	+7.8	65.5	68.4	+2.9	+4.9	+4.2 (0.2 to 8.3)
> 200% FPL	91.0	91.8	+0.8	90.0	92.2	+2.2	-1.4	-1.0 (-3.7 to 1.7)
**Marginal Food Security**								
All Participants	9.4	8.8	-0.6	7.1	6.7	-0.4	-0.2	-0.8 (-1.7 to 0.3)

© 2023 Rook JM et al. *JAMA Health Forum.*

≤ 200% FPL	14.2	12.7	-1.5	11.4	10.8	-0.6	-0.9	-1.6 (-3.1 to -0.1)
> 200% FPL	5.0	5.3	+0.3	5.0	4.7	-0.3	+0.6	+0.5 (-0.9 to 2.0)
**Low Food Security**								
All Participants	7.4	5.2	-2.2	5.9	4.9	-1.0	-1.2	-0.6 (-1.3 to 0.1)
≤ 200% FPL	12.4	8.7	-3.7	12.2	11.2	-1.0	-2.7	-1.5 (-3.0 to -0.0)
> 200% FPL	2.8	2.2	-0.6	2.9	1.8	-1.1	+0.5	+0.3 (-0.5 to 1.1)
**Very Low Food Security**								
All Participants	4.8	3.3	-1.5	4.9	4.0	-0.9	-0.6	-0.4 (-0.9 to 0.0)
≤ 200% FPL	8.7	6.3	-2.4	10.9	9.7	-1.2	-1.2	-1.1 (-2.2 to -0.0)
> 200% FPL	1.2	0.7	-0.5	2.0	1.2	-0.8	0.3	+0.2 (-0.3 to 0.6)

[a] Models utilize an ordinal logistic regression and adjust for age, sex, race/ethnicity, highest household educational attainment, household employment status, chronic disease burden, anxiety or depression diagnosis, health insurance, number of adults, rurality, receipt of SNAP benefits. [b] Difference-in-differences estimates are calculated by subtracting the adjusted during- vs pre-ECTC probability difference for ECTC-ineligible households from that for ECTC-eligible households. [c] All adults: N=39,479; low-income adults: N=14,670; middle- and upper-income adults: N=24,809. [d] Federal Poverty Line determined from 2020 Census Bureau thresholds (eTable 1).

Abbreviations: ECTC – Expanded Child Tax Credit; pp – Percentage point; Diff-in-Diff – Difference-in-Difference; FPL – Federal Poverty Line; <200% FPL – Low-Income Households; >200% FPL – Middle- and Upper-Income Household

© 2023 Rook JM et al. *JAMA Health Forum.*

eFigure 1: Frequency of adults reporting excellent or very good health, National Health Interview Survey, January 2019 to December 2021 [a,b]

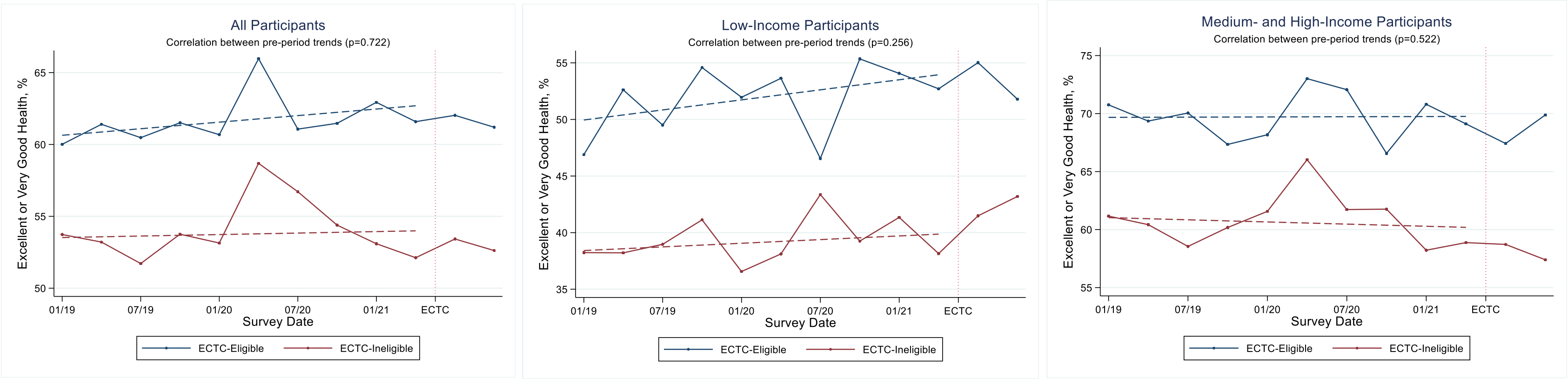

Figure 1B: Frequency of adults reporting food security, National Health Interview Survey, January 2019 to December 2021 [a,b]

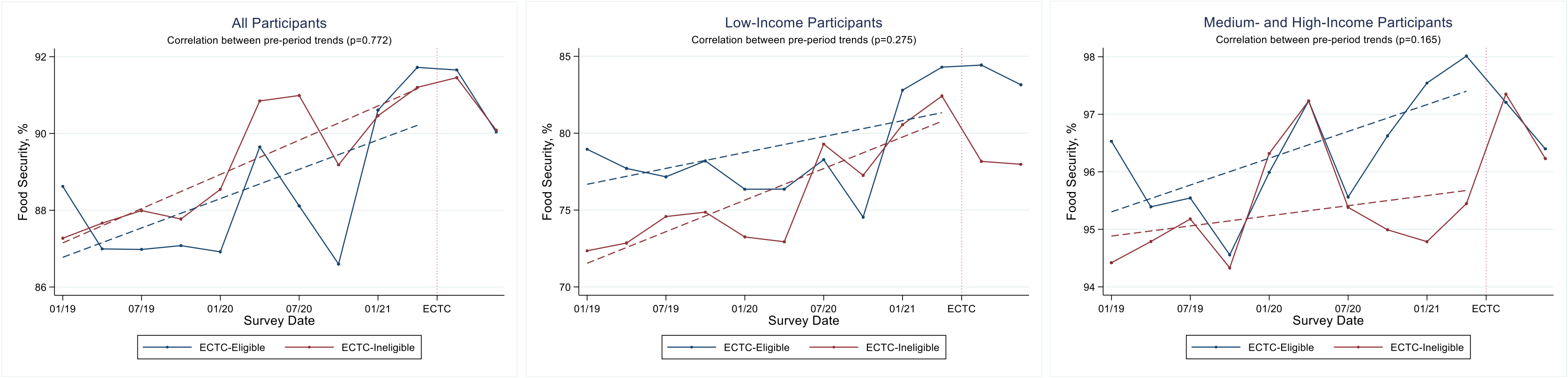

[a] *P*-value refers to a comparison of the pre-ECTC trends between ECTC eligibility groups. A significant value (P<0.05) indicates a difference in the pre-period trends between eligibility groups. The dashed lines represent the pre-trends for the ECTC-eligible group (blue) and the ECTC-ineligible group (red). [b] The red dotted vertical line indicates the start of ECTC monthly payments in July of 2021.

Abbreviations: ECTC – Expanded Child Tax Credit; Low-Income – ≤200% FPL; Middle- and Upper-Income – >200% FPL; FPL – Federal Poverty Line

© 2023 Rook JM et al. *JAMA Health Forum.*
