# Supplementary material for: Changes in Self-Reported Adult Health and Household Food Security With the 2021 Expanded Child Tax Credit Monthly Payments
Source: JAMA Health Forum. 2023 Jun 24;4(6):e231672. doi: 10.1001/jamahealthforum.2023.1672 (PMC10290752; doi:10.1001/jamahealthforum.2023.1672)
Supplement: Supplement 2. — Data sharing statement [file jamahealthforum-e231672-s002.pdf]

Data Sharing Statement

Rook. Changes in Self-Reported Adult Health and Household Food Security With the 2021 Expanded Child Tax Credit Monthly Payments. *JAMA Health Forum*. Published June 24, 2023. doi:10.1001/jamahealthforum.2023.1672

Data
Data available: Yes
Data types: Other (please specify)
Additional Information: Data for the 2019 to 2021 National Health Interview Surveys are already publicly available at the National Center for Health Statistics.
How to access data: https://www.cdc.gov/nchs/nhis/index.htm
When available: beginning date: 12-22-2022

Supporting Documents
Document types: None

Additional Information
Who can access the data: All data used for this study is already publicly available.
Types of analyses: All data used for this study is already publicly available.
Mechanisms of data availability: All data used for this study is already publicly available.
Any additional restrictions: None
